# Resistance of the Dental Pulp to the Action of Arsenious Acid

**Published:** 1868-09

**Authors:** 


### RESISTANCE OF THE DENTAL PULP TO THE ACTION OF ARSENIOUS ACID.

Editors Register:—In your July number a correspondent, who signs himself M. McCarty, states in a very concise and clear manner, a fact that must have come under the observation of every Dentist who has had much experience in devitalizing dental pulps with arsenious acid.

Dr. McCarty states as the conclusion to which he has arrived, after some investigation, that all pulps whose surfaces are freshly exposed or abraded, are acted upon immediately on the application of the acid, while those pulps that have been exposed for some time, resist the action of that agent. The reason which the Doctor advances for this latter result, viz: that the pulp, in all such cases, throws out, by a vital process, a granulated, protecting covering, which, being itself unaffected by the action of the devitalizing agent, defends the pulp from that action, it is not my intention to discuss, although it might well be questioned, whether the explanation would account for all, or even a majority of the cases of resistance of the pulp to devitalization.

I well remember hearing Prof. Taft discuss the question of the devitalization of the dental pulp with arsenious acid; and his theory as to its action was, that it produced death by entering into the circulation, and in no other way. And in this he was supported by his associate professors, and by other gentlemen high up in the profession. I remember too, his speaking of the difficulty he sometimes had to kill the pulp, even with repeated applications of arsenious acid, and if I remember rightly, he did not attribute this to the acid not having fair play, but rather to an extraordinary vital resistance, an unusual tenacity of life in the pulp itself, a result of the peculiar constitution of the patient, whose general system would not, perhaps, be overcome by an ordinary killing dose of arsenic.

Granting, however, that Dr. McCarty's *protection* theory is correct, I think that the result which he aims at may be attained by a simpler method, differing from his rather in degree than in kind. Supposing the operator to be using that preparation of arsenic known as "nerve paste," it is admitted that but a very small quantity of this entering into the circulation of the pulp is sufficient for its devitalization. Dr. McCarty recommends that the protecting surface be removed in order to clear the way for the action of the poison. Now, when it is remembered how tender the pulp surface is—how exceedingly susceptible it is to the slightest thermal changes, so much so that the application of a cold instrument to it will often cause intense pain, and how delicate must be the manipulation to avoid tormenting the patient beyond endurance, it will be readily seen what difficulties must necessarily surround this operation, not to mention its tediousness. Why not simply take a portion of the paste upon the sharp point of an instrument, and thus *puncture* the pulp? The pain will be sharp, but short—the protection, if there be any, will be readily overcome, and the operator will have the satisfaction of knowing that the devitalizing agent has been placed just where it will have its best effect. It seems to me that this course would save time and trouble to the operator, and pain to the patient, to whom it would be much more acceptable than the Doctor's plan, just as the short, sharp work of the bullet would be more acceptable to the condemned than the lingering process of strangling with a rope.

EMERY.

